# Plasmon induced thermoelectric effect in graphene

**DOI:** 10.1038/s41467-018-07508-z

**Published:** 2018-12-05

**Authors:** Viktoryia Shautsova, Themistoklis Sidiropoulos, Xiaofei Xiao, Nicholas A. Güsken, Nicola C. G. Black, Adam M. Gilbertson, Vincenzo Giannini, Stefan A. Maier, Lesley F. Cohen, Rupert F. Oulton

**Affiliations:** 1 Blackett Laboratory, Imperial College, Prince Consort Road, London, SW7 2BZ UK; 2 Department of Materials, University of Oxford, Parks Road, Oxford, OX1 3PH UK; 3 National Physical Laboratory, Hampton Road, Teddington, Middlesex TW11 0LW UK; 4 Instituto de Estructura de la Materia (IEM-CSIC), Consejo Superior de Investigaciones Científicas, Serrano 121, 28006 Madrid, Spain; 5 Chair in Hybrid Nanosystems, Nanoinstitut München, Faculty of Physics Ludwig-Maximilians-Universität München, 80799 München, Germany

## Abstract

Graphene has emerged as a promising material for optoelectronics due to its potential for ultrafast and broad-band photodetection. The photoresponse of graphene junctions is characterized by two competing photocurrent generation mechanisms: a conventional photovoltaic effect and a more dominant hot-carrier-assisted photothermoelectric (PTE) effect. The PTE effect is understood to rely on variations in the Seebeck coefficient through the graphene doping profile. A second PTE effect can occur across a homogeneous graphene channel in the presence of an electronic temperature gradient. Here, we study the latter effect facilitated by strongly localised plasmonic heating of graphene carriers in the presence of nanostructured electrical contacts resulting in electronic temperatures of the order of 2000 K. At certain conditions, the plasmon-induced PTE photocurrent contribution can be isolated. In this regime, the device effectively operates as a sensitive electronic thermometer and as such represents an enabling technology for development of hot carrier based plasmonic devices.

## Abstract (web summary)

The photoresponse of graphene-based photodetectors is dominated by photovoltaic and photothermoelectric effects. Here, the authors leverage strongly localised plasmonic heating of graphene carriers to detect a second photothermoelectric effect occurring across a homogeneous channel in the presence of an electronic temperature gradient.

## Introduction

**Fig. 1:**
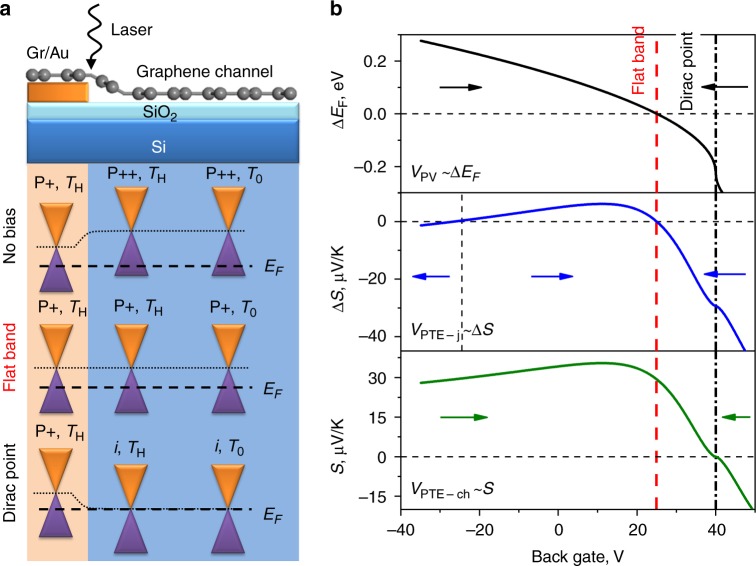
Photocurrent generation mechanisms at graphene/metal interface. **a** Schematic of a graphene/Au interface and associated band diagrams for various gating conditions. The black dash line represents the Fermi level and the black dotted line represents the Dirac point of the graphene. The illumination is localized at the graphene/Au interface resulting in a higher local carrier temperature (*T*_H_) compared to the bath temperature (*T*_0_). **b** Calculated gate voltage dependence of Fermi level difference (top) and Seebeck coefficient difference (middle) between the Gr/Au and Gr/SiO_2_ areas and gate voltage dependence of Seebeck coefficient for the graphene channel (bottom). The contributing current directions are indicated by arrows. Vertical red (dash) and black (dash–dot) lines correspond to the flat band and Dirac points, respectively. Seebeck coefficients are calculated based on the conductivity model at *T* = 300 K. See Supplementary Discussion 1 for the calculation details

Graphene’s optoelectronic response is governed by hot carrier effects due to slow electron-lattice relaxation that results in quenched electron cooling and leads to photogenerated carriers being thermally decoupled from the crystal lattice^1–5^. The hot carrier regime gives rise to a strong photothermoelectric (PTE) effect that can under certain circumstances dominate over the photovoltaic (PV) effect^1,3–6^. A strong PTE effect, which we will refer to as the PTE junction (PTE-j) effect, has been reported for graphene devices at the junction of monolayer and bilayer graphene^4^ at the junction between regions of graphene with different Fermi energies (*E*_F_), such as supported/suspended graphene interfaces^6^ and at graphene p–n junctions with buried split-gates^2^ and top-gate control^1,5^. Graphene–metal interfaces are another well-known example of junctions with different *E*_F_. In this case, the *E*_F_ of graphene on top of or below a metal pad is shifted compared to the metal-free graphene channel due to the difference in work functions of the materials (Fig. 1a)^7,8^. In all of these graphene devices, the PTE voltage is generated at the junction (PTE-j) and is driven by the difference in graphene’s Seebeck coefficients ((*S*_1_ – *S*_2_) = Δ*S*) either side of the junction (e.g. a gate-tunable graphene channel and a pinned graphene/metal region) through $V_{{\mathrm{PTE - j}}} = \left( {S_1-S_2} \right)\Delta T_{\mathrm{e}}^{\mathrm{j}}$, where $\Delta T_{\mathrm{e}}^{\mathrm{j}}$ is the electron temperature increase within the junction after photoexcitation^3^. The dependence of the PTE-j current on Δ*S* results in multiple photocurrent sign reversals over a gate voltage sweep due to the nonmonotonic dependence of *S*_1,2_ on *E*_F_ (Fig. 1b for the case of the graphene–metal junction). This is distinct from the PV effect, in which the photovoltage, *V*_PV_ is related to Δ*E*_F_ and a single sign change is observed at the flat-band point (Fig. 1b top panel), allowing the PTE-j effect to be identified. Numerous studies of metal–graphene–metal photodetectors have demonstrated the presence of a single sign reversal far from the Dirac point, which is typically assigned to the flat-band condition^9–13^. However, it is impossible to distinguish the PV and PTE-j contributions in this regime since both effects have equal signs (e.g. Fig. 1b in the region of −20 to 40 V).

In this paper, we report an additional but distinct PTE contribution, where a global electronic temperature difference is established across the device channel ($\Delta T_{\mathrm{e}}^{{\mathrm{ch}}}$) itself, which we will refer to as the PTE channel (PTE-ch) effect to distinguish it from the known PTE-j effect. In contrast to the PTE-j effect, the PTE-ch voltage is driven by $\Delta T_{\mathrm{e}}^{{\mathrm{ch}}}$ according to $V_{{\mathrm{PTE - ch}}} = S\Delta T_{\mathrm{e}}^{{\mathrm{ch}}}$, where *S* is the Seebeck coefficient of the graphene channel. By inspection of Fig. 1b (bottom panel), it is clear that *V*_PTE-ch_ should exhibit a single sign change at the Dirac point of the graphene in the channel, which is not present in either *V*_PTE-j_ or *V*_PV_. This PTE-ch effect is reminiscent of the conventional (i.e. not photon-induced) thermoelectric (TE) experiments where a heater is an internal part of the device contacts^14^. The electronic temperature gradient across the channel may, in principle, be present when only one side of the metal–graphene–metal device is illuminated. However, studies of these graphene photodetectors to date have typically reported carrier temperature increases of just a few degrees Kelvin or less^5,15^ so that the contribution from *V*_PTE-ch_ was negligible compared to *V*_PV_ or *V*_PTE-j_.

Here we utilize an asymmetric electrical contact arrangement where one electrode incorporates plasmonic nanostructures to generate an electronic temperature gradient across a graphene channel. As we have previously demonstrated in ref. ^16^, under plasmon excitation of a metal nanostructure, the highly localized and strongly enhanced electromagnetic fields around the nanostructure drastically improve photo-absorption in the nearby graphene, resulting in efficient and localized carrier heating in the graphene. Therefore, an asymmetric plasmonic contact geometry is expected to produce a large electronic temperature gradient across the graphene channel needed to create and observe a strong PTE-ch effect. Moreover, as the gate-dependence of *V*_PTE-ch_ is distinct from that of *V*_PTE-j_ and *V*_PV_, we can expect to identify and isolate this mechanism from the other contributions at the graphene/contact junctions, when the flat band and Dirac points occur under distinct gating conditions.

## Results

### Photoresponse of asymmetric plasmonic contacts

**Fig. 2:**
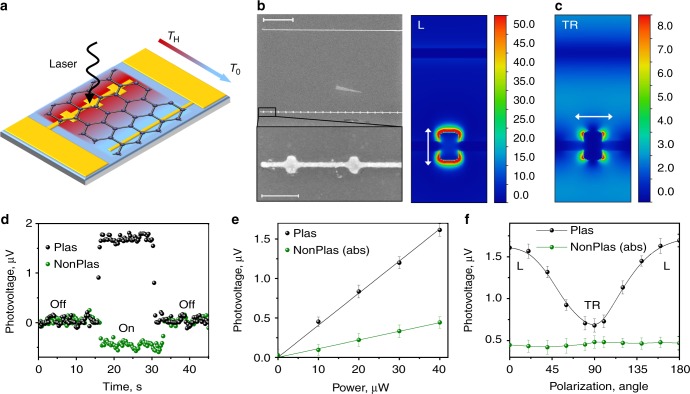
Photoresponse of a metal–graphene–metal photodetector with asymmetric plasmonic contacts. **a** Schematic of the graphene device with plasmonic and nonplasmonic contacts. Under optical excitation of the plasmonic contact, the local electronic temperature rises to *T*_H_ compared to the bath temperature (*T*_0_) resulting in the temperature gradient established across the contacts as indicated by the arrow. **b** Scanning electron microscopic image of the device (upper panel, scale bar is 2 µm) and graphene/Au nanostructures (lower panel, scale bar is 300 nm). **c** Calculated electromagnetic field distributions for longitudinal (L) and transverse (TR) polarization at 740 nm. **d**–**f** Photovoltage generated at 740 nm as a function of time (**d**), laser power (**e**) and polarization (**f**) for plasmonic (Plas) and nonplasmonic (NonPlas) contacts. For ease of comparison, the photovoltage generated for the nonplasmonic contact is presented in absolute values (abs). The measurements in **d**, **e** were performed under L polarization. The laser power in **d**, **f** is fixed at 40 µW. The error bars are the standard deviation in the measurements

The geometry of our graphene photodetector is similar to that of typical metal–graphene–metal detectors previously reported^8,10,11,13^ but with the key difference that one of the electrodes comprises resonant nanostructures (the plasmonic contact), as shown in Fig. 2a. This type of plasmonic nanostructure is chosen due to its relative ease of fabrication and wavelength tunability (see Supplementary Discussion 2 and Supplementary Figures 1, 2 for more information on the contact design). Figure 2b shows an scanning electron microscopic (SEM) image of an exemplary graphene device with a channel length of 5.5 µm. The contact length is 10 µm, which in the case of the plasmonic contact permits incorporation of 19 plasmonic nanoparticles (NPs) arranged with a pitch of 500 nm. The target dimensions of each NP are 140 × 90 nm^2^; however, at these scales the fabricated particles appear slightly elliptical, as evident from Fig. 2b. The plasmonic NPs support longitudinal (L) and transverse (TR) resonances when excited with light polarized along the length and width of the NP, respectively, as shown in Fig. 2c^17,18^. In our study, we utilize the L resonance, which occurs near a wavelength of 700 nm. It should be noted that the results presented in this study are obtained for several devices. Please refer to Supplementary Discussion 3 and Supplementary Figure 3 for more details on the various devices used in this study.

We first perform standard photocurrent microscopy by scanning a polarized elliptical laser beam (1 × 20 µm^2^) over the device at illumination wavelength 740 nm, close to the NP L-resonance (with the beam long axis aligned with the electrode long axis, see Methods for details). As the channel length is substantially greater than the width of the laser beam, the separate photovoltage responses of each electrode can be clearly resolved (Fig. 2d). Consistent with previous studies of metal–graphene–metal photodetectors^8,10,11^, each electrode generates a photovoltage of opposite polarity. However, the photoresponse of the plasmonic contact is strongly increased compared to the normal (nonplasmonic) electrode (Fig. 2e). As such, in contrast to previous studies with symmetric electrodes made of the same metal, the photovoltage generated by this device is non-zero under uniform illumination. The influence of the plasmonic contact on the photovoltage is confirmed by the strong $\cos ^2\theta$ dependence on polarization angle (*θ*), characteristic of any dipole antenna (Fig. 2f). In contrast, the photovoltage from the nonplasmonic contact exhibits negligible polarization dependence. Note that the photovoltage from the plasmonic electrode at *θ* = 90° remains above that of the nonplasmonic electrode due to the presence of a weak TR resonance at 740 nm (Fig. 2c).

**Fig. 3:**
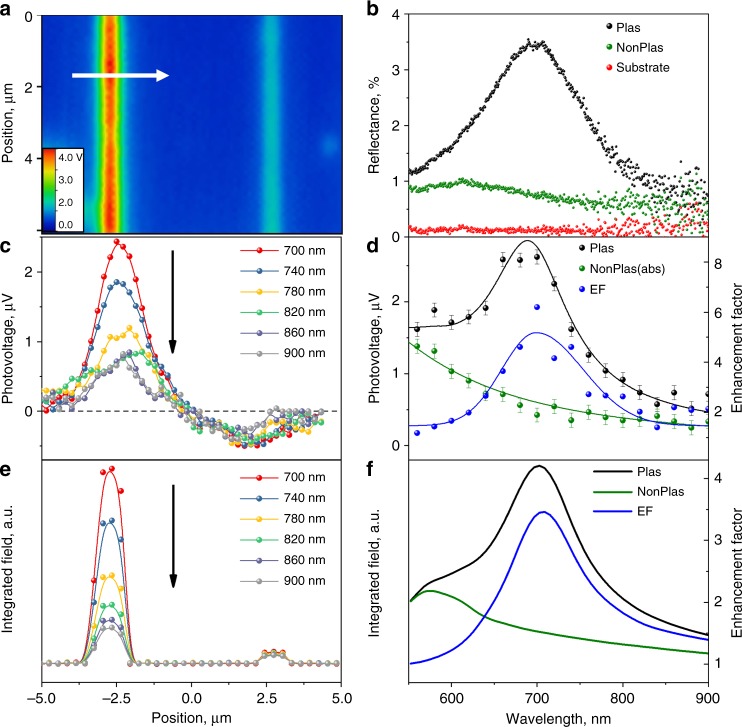
Plasmon-enhanced graphene photoresponse. **a** Dark field microscopic image of the graphene device with plasmonic (left) and nonplasmonic contacts (right). **b** Corresponding dark field reflection spectra. **c** Photovoltage line scans across the device in the direction indicated by the arrow in **a** taken at different excitation wavelengths. **d** Wavelength dependence of the photovoltage (absolute value) from the plasmonic and nonplasmonic contacts. Enhancement factor is determined as following *V*_Plas_/*V*_NonPlas_. The photovoltage measurements are performed with an elliptical laser spot with 40 µW power. **e** Calculated line scan of the integrated electromagnetic field. **f** Calculated wavelength dependence of the integrated electromagnetic field and enhancement factor (Plas/NonPlas). The error bars are the standard deviation in the measurements

The presence of plasmonic NPs at the contact is accompanied by enhanced scattering. This is evident from the dark field microscopic image shown in Fig. 3a. Meanwhile, the dark field scattering spectra highlight the strong plasmonic resonance of the NP L mode at 700 nm (Fig. 3b). The photovoltage measurements were repeated at various excitation wavelengths in the range from 500 to 900 nm. Figure 3c depicts photovoltage line scans across the device at various excitation wavelengths, while Fig. 3d shows the resulting photovoltage spectrum obtained by plotting the maximum absolute value of the photovoltage for the plasmonic and nonplasmonic contacts. The plasmonic contact’s photovoltage spectrum demonstrates a clear resonance similar to the dark field spectrum, while the nonplasmonic electrode’s photovoltage response shows a monotonic decrease with the excitation wavelength. These experimental results are also in remarkably good agreement with the calculations of the integrated electromagnetic field, as shown in Fig. 3e, f. Therefore, we attribute the observed photoresponse improvement to electromagnetic field enhancement around the plasmonic NPs. It is striking that the presence of NPs with quite low fill factor of ~20% can increase the photoresponse 5 times, although this is entirely consistent with the calculation results.

### Mechanism of plasmon-induced photoresponse enhancement

To separate the physical mechanisms contributing to the photovoltage generation in our device, we perform gate-dependent photovoltage measurements using the substrate as a back gate. Gating affects only the *E*_F_ of the channel graphene^7^, changing its TE properties and the band-bending conditions at the graphene/metal contact junction. Of particular interest is the flat-band condition, where both junction effects, namely PV and PTE-j, are cancelled. Thus, under the flat-band condition, the only active contributing effect is the plasmon-induced PTE-ch effect, as shown in Fig. 1b. The photovoltage generation mechanisms present at various gating conditions are summarized in Table 1.

**Table 1:** Summary of contributing effects under different gate bias: ✓—the effect is active and ✕—the effect is cancelled

Effect	As prepared	Flat band	Dirac point
	*V*_BG_ = 0	*V*_BG_ = 25 V	*V*_BG_ = 40 V
PV	✓	✕	✓
PTE-j	✓	✕	✓
PTE-ch	✓	✓	✕

**Fig. 4:**
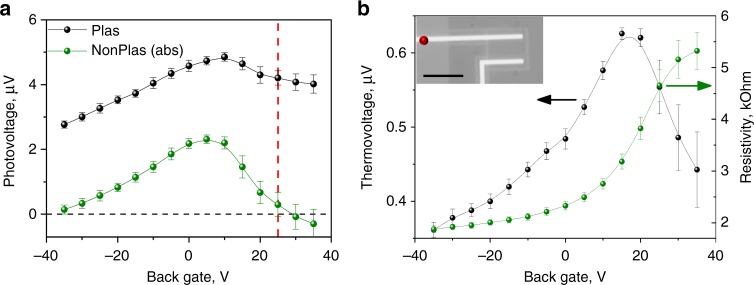
Gate-dependent photovoltage and thermoelectric response. **a** The photovoltage response of plasmonic and nonplasmonic (absolute value) contacts. Photovoltage measurements are performed with an elliptical laser spot at 700 nm with 40 µW power under longitudinal polarization. The vertical dash line represents the flat-band condition. It should be noted that a direct comparison of the photovoltage generated for the plasmonic and nonplasmonic contacts in terms of absolute values is complicated owing to contact imperfections (Supplementary Discussion 3). **b** The thermoelectric voltage and square resistance of a representative FET device. Inset: greyscale optical microscopic image of the device with the laser position marked by a red dot. Scale bar is 10 µm. Measurements are performed with a focussed laser spot at 750 nm and 3 mW power. The error bars are the standard deviation in the measurements

Figure 4a shows a comparison of the gate-dependent photovoltage response for the plasmonic and nonplasmonic contacts under L polarization. Interestingly, the nonplasmonic contact demonstrates a polarity change at ~25 V. In contrast, the photovoltage signal at the plasmonic contact remains strongly positive over the entire gate voltage range. Since the Dirac point is expected at ~40 V (see detailed discussion below), the sign change at ~25 V can be attributed to the flat-band condition. This observation is consistent with previous studies of graphene/metal junctions, where a sign reversal is frequently observed before the Dirac point^9–12^. As such, we can infer that the graphene doping above the metal contact and in the channel are equal at the sign-change point. Moreover, the position of the sign change relative to the Dirac point indicates a p-type doping of the graphene on top of the gold metal electrode that is lower than the p-type doping in the graphene channel. From the carrier density *n* = *ε*_0_*ε*_SiO2_(*V*_g_ − *V*_D_)/*et*, where *t* is the oxide thickness and *V*_g_ and *V*_D_ are gate voltage and the Dirac point position, respectively, we deduce an *E*_F_ = ℏ*ν*_F_(*πn*)^1/2^ of 0.25 and 0.4 eV for the graphene over the gold electrode and SiO_2_, respectively. The higher p-doping in the graphene channel can be explained by SiO_2_-induced charge transfer^19^. The doping *E*_F_ values are consistent with the results of Raman spectroscopy analysis, discussed in Supplementary Discussion 4 and Supplementary Figures 4, 5.

It is important to understand the gate dependence of the conventional TE effect to directly compare to the PTE-ch effect, which has the same physical origin. Therefore, we analyse the gate-dependent resistance and TE properties of a representative two-terminal graphene test device with a 5 × 8 µm^2^ channel, shown in Fig. 4b. We introduce localized heating by the focussed laser spot of 1 µm and 3 mW power positioned over one of the contacts at a distance (~15 µm) away from the photoactive area of the graphene junction. By heating one of the contacts, a fixed temperature difference Δ*T* between the source and drain electrodes is established, generating a thermovoltage *V*_TE_ = *S*Δ*T*. The gate-dependent thermovoltage measured across the device directly reflects the variation of the Seebeck coefficient for the channel graphene^1^ and thus provides a characteristic footprint of the TE and PTE-ch effects. As shown in Fig. 4b, the thermovoltage exhibits a nonmonotonic behaviour that peaks close to the Dirac point, as previously reported in ref. ^20^. The sign of the thermovoltage indicates the sign of the majority charge carrier and is expected to change from positive to negative, as the gate voltage crosses the Dirac point. However, the observed thermovoltage remains positive, indicating that the Dirac point is not reached for this range of gate bias values, consistent with the resistance measurements shown in Fig. 4b (right axis) (higher bias conditions are not practical due to dielectric breakdown). Nevertheless, using the conductivity model (see Supplementary Discussion 5 and Supplementary Figure 6) we can estimate the Dirac point to be about ~40 V.

Next, we compare the overall trends observed for the plasmonic and nonplasmonic contacts to the TE response of the test device. It is evident that the gate-dependent photovoltage of the plasmonic contact demonstrates a quite similar behaviour to the thermovoltage (Fig. 4). In particular, no sign change is observed for either case over the range of gate voltages used. However, the non-zero photovoltage signal at the flat-band condition (~25 V), where the PV and PTE-j effects are effectively cancelled, and indeed the overall vertical offset in the plasmonic photovoltage compared to the nonplasmonic electrode, suggest that an additional, more dominant, photovoltage generation mechanism is present for the plasmonic electrode, which we ascribe to the plasmon-induced PTE-ch effect. Notably, the contribution of the direct laser heating of the metal contact towards the plasmonic detector photoresponse is negligible in our photovoltage experiment, please refer to Supplementary Discussion 7 and Supplementary Figure 8.

### Plasmon-induced hot carrier temperature

The PTE-ch effect arises due to the carrier temperature difference between two contacts and thus provides an opportunity to analyse the plasmon-induced carrier temperature in graphene. Since the hot carrier population established in graphene after photoexcitation can be described by a thermal distribution with a well-defined hot carrier temperature *T*_C_^3,21^, the voltage generated due to the PTE-ch effect for the plasmonic contact can be expressed as

1 $$V_{{\mathrm{PTE - ch}}} = - \mathop {\int }\nolimits_{T_0}^{T_{\mathrm{H}}} S\left( {T_{\mathrm{C}}} \right){\mathrm{d}}T_{\mathrm{C}},$$

where *T*_H_ and *T*_0_ are the carrier temperatures at the plasmonic and nonplasmonic contacts, respectively. The hot carriers generated in the vicinity of the plasmonic contact diffuse into the graphene layer and create an electronic temperature gradient. The profile of the temperature gradient is discussed further in Supplementary Discussion 8 and Supplementary Figure 9 using a simple one-dimensional differential heat transport equation. While the temperature profile is sensitive to the input parameters in the model, the results suggest that the carrier temperature at the nonplasmonic contact *T*_0_ remains at *T*_bath_ ~ 300 K for a wide range of cooling lengths of 0.2–1 µm, which are typical values for graphene observed at room temperature^22,23^.

Following ref. ^14^, we integrate Eq. (1) to obtain

2 $$T_{\mathrm{H}} = \sqrt {(T_0)^2 + 2(\left| {V_{{\mathrm{PTE - ch}}}/k} \right|)} ,$$

where the temperature-independent coefficient *k* is related to Seebeck coefficient through *k* = *S*/*T*. The graphene Seebeck coefficient can be estimated from the electrical conductivity measurements using the simplified Mott relation $S_{{\mathrm{Mott}}} = - \frac{{\pi ^2k_{\mathrm{B}}^2T}}{{3e}}\frac{1}{\sigma }\frac{{{\mathrm{d}}\sigma }}{{{\mathrm{d}}E_{\mathrm{F}}}}$, which provides a good approximation for a range of temperatures and carrier densities^14,20,24,25^. Although this expression is only valid under limited circumstances, it has been successfully employed to describe the PTE effect in graphene junctions^1,3–6,12,23,26,27^. Please refer to Supplementary Discussion 6 and Supplementary Figure 7 for further details as well as the results of the calculation of the *k* coefficient.

The photovoltage measurements discussed in Figs. 2–4 represent the time-averaged signal *V*_ph_ resulting from a laser pulse train. To access the instant electronic temperature generated by a single ultrafast laser pulse, the peak photovoltage should be restored taking into account the laser duty cycle according to *V*_peak_ = *V*_ph_/*ν*_0_*τ*_r_, where *ν*_0_ is the laser repetition rate of 80 MHz and *τ*_r_ is the average photovoltage pulse width related to the device response time (Supplementary Discussion 9 and Supplementary Figure 10.).

**Fig. 5:**
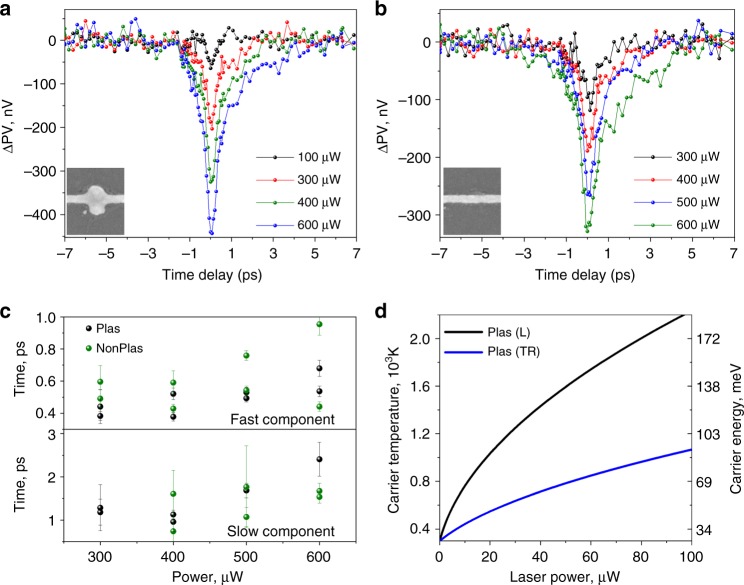
Plasmon-induced carrier heating. **a**, **b** Probe-induced photovoltage at plasmonic and nonplasmonic contacts as a function of pump–probe pulse delay time. Probe at wavelength of 750 nm and 100 µW power, pump at a wavelength of 650 nm and power as indicated; both beam spots have elliptical shape. **c** Response time extracted from the bi-exponential decay of the dip in **a**, **b** as a function of the pump power. Error bars reflect the estimated standard deviation of the fit coefficients. **d** Power dependence of the carrier temperature and carrier energy in graphene at the plasmonic contact under longitudinal (L) and transverse (TR) polarizations

To determine the device response time, we perform time-resolved photovoltage measurements using a pump–probe technique (see Methods for more details). In this experiment, we study the photovoltage as a function of the time delay Δ*t* between a pump pulse at a wavelength of 650 nm and a probe pulse at a wavelength of 750 nm for a fixed probe power of 100 µW and a range of incident pump powers. Notably, the selected wavelengths are close to the L-mode resonance to facilitate plasmon excitation. Since the probe beam is modulated, the measured signal can be considered as the probe-induced photovoltage. Figure 5a, b show the time-resolved photovoltage traces obtained for local excitation of each contact as a function of pump power, after subtraction of the baseline signal (given by the signal far away from zero-time delay). The pump pulse affects the probe-induced photovoltage response only near zero-time delay and results in a sharp photovoltage dip due to saturation of photocarrier generation at the probe energy. In graphene, this saturation regime is determined by pump-induced Pauli blocking of electronic states and is observed when the photogeneration rate is comparable to the energy relaxation and recombination rates^28,29^. Thus increased pump power (higher generation rate) results in greater saturation, which is clearly observed in the experimental results for both electrodes (Fig. 5a, b). Notably, the plasmonic contact exhibits a larger saturation amplitude compared to the nonplasmonic contact, which is entirely consistent with higher photoresponsivity observed for this electrode (Fig. 3d).

The width of the dip observed for the overlap condition corresponds to the carrier relaxation time required for the graphene device to return to equilibrium. The photocarrier dynamics in graphene are well described by a bi-exponential decay with a short sub-picosecond (fast) component immediately after the temporal overlap of the pump/probe pulse followed by a longer picosecond (slow) component^15,30^. Accordingly, we fit the experimental photovoltage data with two-sided bi-exponential decay to extract relaxation times, the results of which are shown in Fig. 5c. The extracted fast and slow relaxation times both exhibit a slight increase with pump power. This is in agreement with previous studies^23,30^ where this observation was attributed to the accumulation of photocarriers in the high-field region leading to a longer relaxation time required.

Using the extracted decay times and amplitudes of the fast and slow components, we deduce an average photovoltage pulse width of *τ*_r_ = 0.85 ± 0.13 ps (Supplementary Discussion 9), allowing *V*_peak_ to be estimated from the experiments of Figs. 2–4. The carrier temperature at the nonplasmonic contact is assumed to be *T*_0_ ~ *T*_bath_ ~ 300 K for a cooling lengths of 0.2–1 μm (Supplementary Discussion 8). Taking into account the value of *τ*_r_ and assuming a hot carrier diffusion coefficient of 5500 cm^2^ s^−1^ for chemical vapour deposited (CVD) graphene^31^, the estimated diffusion length is of ~700 nm substantially less than the channel length of 5.5 μm, further supporting our assumed value for *T*_0_. As discussed earlier, of particular interest is the flat-band condition occurring at *V*_g_ ~ 25 V, where the PV and PTE-j effects are cancelled. At this gate bias, we determine *k* ≈ 63 nV K^−2^ (Supplementary Discussion 6). Taking into account the plasmonic contact’s response at 25 V and its linear power dependence, we can finally estimate the carrier temperature according to Eq. (2), which is shown in Fig. 5d. Under illumination with longitudinal polarization, resonance conditions corresponding to the NP L mode can be reached leading to carrier temperatures of ≈2200 K in the vicinity of the plasmonic contact. Under TR polarization, we estimate a carrier temperature of the order of 1000 K. The extracted temperatures are in reasonable agreement with our previous studies of ultrafast carrier heating in graphene/plasmonic NP complexes, where carrier temperatures of 1500 K were estimated^16^. In the latter work, this value was derived using differential reflection measurements and a phenomenological two-temperature model taking into account electronic heating efficiency of graphene. In this respect, the present work provides a more direct way of measuring the plasmon-induced carrier temperature.

To show that plasmon-induced carrier heating is indeed efficient, we compare our results to typical carrier temperatures observed for graphene p–n junctions, where the PTE-j effect is dominant^1,3^. It has been demonstrated that, under illumination conditions of 3.5 × 10^12^ photons cm^−2^ (equivalent to 100 µW in our experiment), the hot carrier temperature can reach ~200 K over the initial bath temperature of *T*_bath_ ≈ 10 K^32^. In stark contrast, in our experiments, under same illumination conditions, plasmonic amplification results in carrier temperatures as high as ~2200 K. High electronic temperatures aided by plasmonic enhancement sets out the potential for hot carrier-driven photochemistry applications. Moreover, as a sensitive electron (or hole) thermometer, the mechanism and device geometry we demonstrate here can be utilized for optimization of hot carrier generation for the growing field of plasmonic-driven hot carrier applications.

In terms of the detectivity of our devices, in the presence of plasmonic nanostructures, the device responsivity at the resonance wavelength is strongly enhanced, achieving a ~5 times improvement over conventional devices, corresponding to 0.125 mA W^−1^ with a device resistance of 1 kOhm. Nevertheless, this is about an order of magnitude lower than the best-reported responsivity to date (6.1 mA W^−1^) for two-terminal metal–graphene photodetectors using high-mobility and low-defect exfoliated graphene^13,33^. The mobility of our devices is measured to be in the range of 700–1000 cm^2^ V^−1^ s^−1^. Notably, significant improvement could be achieved by encapsulation with exfoliated or CVD-grown hBN layers^34–36^, which typically results in the material with mobility in the range of 25,000–100,000 cm^2^ V^−1^ s^−1^ and higher Seebeck coefficients by a factor of 2^37^. Consequently, the responsivity of our device geometry could be significantly improved.

## Discussion

In summary, we have demonstrated that plasmonic nanostructures can be utilized as part of an asymmetric contact arrangement in a photodetector device to generate a strong electronic temperature gradient across the graphene channel and thus drastically enhance a PTE current generated across the device channel. This effect is distinct from the typical PTE effect generally observed at graphene p–n junctions. By studying the gate-dependent photoresponse, we have identified a regime where this plasmon-induced PTE contribution can be isolated and where our device can operate as an effective electron temperature thermometer. Ultrafast photocurrent studies have shown the device response time of ~2 ps. Consistent with our proposed plasmon-enabled PTE mechanism, we have estimated the local plasmon-induced electronic temperatures to be ~2200 K on picosecond time scales. The demonstrated device geometry can be utilized as a sensitive electron thermometer and also has the potential to contribute to the development of free space compact and ultrafast photodetectors for a range of wavelengths due to plasmon-enabled spectral tunability.

## Methods

### Sample fabrication

The photodetectors were fabricated on a highly conductive Si wafer (p-type) to avoid photogating effects due to absorption in Si. A 90-nm-thick layer of thermal oxide was used as a back gating dielectric. Photolithography technique was used to fabricate microscopic contacts (5 nm Cr, 25 nm Au) followed by electron beam lithography used to define nanocontacts (40 nm Au). CVD graphene purchased from Graphenea was deposited on top of prefabricated contacts using wet transfer technique. Graphene/Cu layers were covered with poly(methyl methacrylate) (PMMA) A4 495 by spin coating at 3500 rpm for 60 s and curing at room temperature overnight. Graphene layer present on the bottom side of the foils was removed using an O_2_-plasma treatment at 100 W during 2 min. The exposed Cu foil was then etched in an ammonium persulphate solution (15 g L^−1^) followed by a thorough rinse in deionized water. The floating PMMA/graphene stack was scooped out using the target substrate. After drying, the sample was annealed at 180 °C for 30 min. PMMA was removed in acetone followed by isopropanol alcohol rinse. The graphene was patterned using photolithography process followed by O_2_-plasma treatment at 50 W for 30 s. The presence of high-quality single-layer material was confirmed by Raman spectroscopy and atomic force microscopy (Supplementary Discussion 4).

### Graphene device characterization

All the measurements were performed under ambient environment and at room temperature conditions. Raman spectroscopy measurements were performed using a confocal scanning Raman microscope (WiTec Alpha300 system) in a backscattering geometry with 532 nm excitation (1 mW) and a ×100 objective (0.9 NA). Dark-field measurements were conducted in a backscattering geometry with illumination from a Nikon Intensilight C-HGFI mercury lamp, where the scattered light was collected using Nikon LU Plan ELWD ×100 (0.80 NA) objective and transmitted via a fibre to a single-photon-counting module (SPCM-AQRH) connected to a gated photon counter (SR400) or a Princeton Instruments spectrometer for microscopic and spectroscopic measurements, respectively. The transport measurements were conducted using a Keithley 2634B semiconductor parameter analyser unit with a two-point measurement for source and drain electrodes.

### Photovoltage measurements

The devices were illuminated with ultrafast laser excitation provided by tunable 80 MHz mode-locked Ti:Sapphire laser with 150 fs pulses. The laser beam was modulated with a mechanical chopper (~416 Hz), while the open-circuit photovoltage signal was detected with a voltage pre-amplifier (SR560) and a lock-in amplifier (SR830). The back-gate bias was applied using a Keithley 2634B unit. For the time-resolved measurements, the initial pulse at 750 nm was split into two optical paths, and part of it was coupled to a Coherent Chameleon Compact OPO to produce 1300 nm signal that was introduced in second-harmonic generation system (Harmonixx-APE) to provide 650 nm excitation. The initial pulse was delayed using a retro-reflector mounted onto a motorized translation stage (Thorlabs PT1-Z8). The two pulses were combined using a beam splitter and focussed on the sample to form overlapping beams. Previously performed time-resolved photocurrent studies of graphene devices typically utilize cross-polarized beam configuration to minimize interference near zero time delay point^30,32,38^. However, in the plasmon-based photodetectors, cross-polarization is not applicable due to high device sensitivity to the beam polarization. To overcome this issue, a two-colour experiment configuration was selected with wavelengths of 650 and 750 nm to be on resonance with plasmonic nanostructures. It should be noted that pulsed excitation is significantly different in nature from continuous wave (CW) excitation. In particular, CW excitation leads to a steady-state temperature as the system equilibrates with time, while pulsed excitation generates a temperature spike of hot carriers that rapidly cool long before the next pulse arrives. Interestingly, at room temperature the device responsivity is the same for both types of excitation (Supplementary Figure 11). This is in agreement with previously observed temperature dependence of graphene photoresponse generated by CW and pulsed excitation^38^. The extended power dependence of the photoresponse for plasmonic contact is presented in Supplementary Figure 12 to demonstrate linear regime of operation.

### Calculation results

Numerical calculations were performed using a finite-difference time-domain method (Lumerical FDTD Solutions). For Fig. 3e, f, the electromagnetic field distribution was integrated over the area of 1 µm^2^ to reproduce illumination conditions. The Gaussian profile has been incorporated through multiplication with $e^{ - \left( {x - x_{\mathrm{L}}} \right)^2/R_{\mathrm{L}}^2}$, where *x*_L_ is the position of the laser spot and *R*_L_ is the laser spot size.

## Electronic supplementary material

Supplementary Information

## Data Availability

The data that support the findings of this study are available from the corresponding author upon request.
