# Supplementary material for: Plasmon induced thermoelectric effect in graphene
Source: Nat Commun. 2018 Dec 5;9:5190. doi: 10.1038/s41467-018-07508-z (PMC6281658; doi:10.1038/s41467-018-07508-z)
Supplement: Supplementary file 1 — Supplementary Information [file 41467_2018_7508_MOESM1_ESM.docx]

**Plasmon induced thermoelectric effect in graphene.**

Viktoryia Shautsova^1,2*^, Themistoklis Sidiropoulos^1^, Xiaofei Xiao^1^, Nicholas A. Güsken^1^, Nicola C. Black^1,3^, Adam M. Gilbertson^1^, Vincenzo Giannini^1,4^, Stefan A. Maier^1,5^, Lesley F. Cohen^1^, Rupert F. Oulton^1^

^1^ Blackett Laboratory, Imperial College, Prince Consort Rd, London SW7 2BZ, UK
^2^ Department of Materials, University of Oxford, Parks Road, Oxford, OX1 3PH, UK
^3^ National Physical Laboratory, Hampton Rd, Teddington, Middlesex TW11 0LW, UK

^4^ Instituto de Estructura de la Materia (IEM-CSIC), Consejo Superior de Investigaciones Científicas, Serrano 121, 28006 Madrid, Spain
^5^ Chair in Hybrid Nanosystems, Nanoinstitut München, Faculty of Physics, Ludwig-Maximilians-Universität München, 80799 München, Germany

# Supplementary Discussion 1. Photocurrent generation mechanisms at graphene/metal interface

Here, we provide calculation details for the Fig. 1 (b) from the main paper text demonstrating different mechanisms involved in photocurrent generation at graphene/metal interface. As discussed in the main paper text, there are three major contributing effects including photovoltaic (PV), photothermoelectric junction (PTE-j) and channel (PTE-ch) effects. For PV mechanism, the generated current is expected to follow Fermi energy difference across the graphene/metal interface $V_{PV} \sim\Delta E_{F} = ( E_{F}(Gr/Au) - E_{F}(Gr/SiO_{2}) )$. For PTE-j effect the generated current is proportional to Seebeck coefficient difference across the junction, namely $V_{PTE-j}= (S(Gr/SiO_{2}) - S(Gr/Au))\Delta T_{e}^{j}$*,* where $\Delta T_{e}^{j}$ is the electron temperature increase within the junction after photoexcitation. The last effect involved is due to channel heating and proportional only to Seebeck coefficient of graphene in the device channel $V_{PTE-ch}= S(Gr/SiO_{2})\Delta T_{e}^{ch}$*,* where $\Delta T_{e}^{ch}$ is the electron temperature difference established across the device channel. To demonstrate the various effects, the channel graphene Fermi energy, $E_{F}(Gr/SiO_{2}),$ and Seebeck coefficient, $S(Gr/SiO_{2})$, are calculated based on conductivity model with Dirac point extracted from experimental resistance measurements (see details in the Supplementary Discussion 4,5). The Fermi energy and Seebeck coefficient of the graphene/Au region are extracted based on flat-band condition, as discussed in the main paper text.

# Supplementary Discussion 2. Contact design

The Lumerical finite-difference time-domain (FDTD) simulation software is utilized to perform optimisation of device design. Carrier heating is proportional to electromagnetic field intensity. Therefore, field enhancement near plasmonic nanoparticle (NP) is considered as a figure of merit for the structure design optimisation. The calculation results are presented in Supplementary Figure 1. Firstly, influence of the NP length on the longitudinal plasmon resonance is studied. As expected, the resonance position is highly sensitive to the NP length resulting in the resonance shift towards longer wavelength with increasing NP length (Supplementary Figure 1a). Notably, resonance at telecom wavelengths can be effectively achieved with a NP length of ~350 nm. To provide a good overlap with available experimental facilities, the resonance at ~800 nm, corresponding to NP length of 150 nm, has been chosen. The next important parameter under consideration is NP period. Whilst a higher number of focusing elements is desirable, under small separation destructive plasmon interference is observed decreasing the field enhancement, as can be seen in Supplementary Figure 1b. Therefore, we have determined 500 nm to be an optimal NP period to provide high NP density, while keeping field enhancement only weakly affected.

(a)

(b)

(d)

(c)

**Supplementary Figure 1.** **Finite-difference time-domain simulation results.** Electromagnetic field enhancement in the vicinity (5 nm) of plasmonic nanoparticles (NPs). (a) Shift of longitudinal resonance with NP length, NP width is 100 nm. (b) Influence of NP period (b), contact width (c) and Cr adhesion layer (d) on NP field enhancement.

Next, the effect of contact integration with NPs is analysed in terms of field enhancement modification (Supplementary Figure 1c, d). Integration of NPs with metallic contacts results in a plasmon resonance that is significantly weakened compared to an individual isolated NP (Supplementary Figure 1b). To keep this effect low, the contact is fabricated with a width of only 40 nm. As the last optimization parameter, we consider the material of the contacts and, in particular, the influence of an adhesion layer. It is clear from Supplementary Figure 1d, that the presence of even 2 nm of a Cr layer results in a significant damping of the plasmon resonance. This effect has been previously observed for Ti adhesion layer as well [1,2]. The contacts thus are fabricated from gold (40 nm in thickness) without any adhesion layer. To improve the contact adhesion, the developed samples after ebl fabrication are exposed to O_2_-plasma for 30s at 50W and 40sccm.


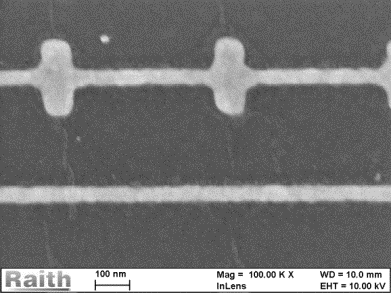

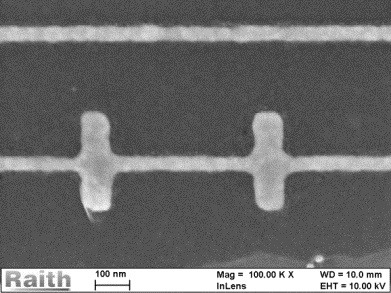

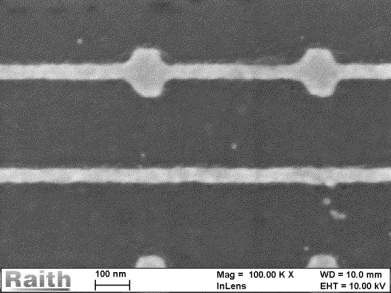


(b)

150 nm

225 nm

300 nm

(a)

**Supplementary Figure 2.** **The tunability of plasmon resonance.** (a) SEM images of the plasmon contacts with varying length of nanoparticles (NPs). (b) FTIR spectra of the contact arrays demonstrating plasmonic resonances as indicated by arrows.

Supplementary Figure 2 shows the tunability of plasmon resonance with varying NP length indicating potential for multi-wavelength photodetection.

# Supplementary Discussion 3. Optical microscopy of the analysed devices

Supplementary Figure 3 shows optical microscopy images of the devices tested in this paper. Due to the high electrostatic sensitivity of graphene devices, operation failure is quite common whilst performing electrical measurements. Furthermore, connections to a chip carrier are made using a wedge bonding technique that utilizes ultrasonic power and force applied to the target substrate contacts. This technique can result in damage of the thin SiO_2_ layer (90 nm) limiting the range of devices available for back gating experiments. Devices 1-3 are typical photodetectors based on asymmetrical plasmonic contacts, as can be seen from optical microscopy images where the plasmonic contact appears to be much darker. Device 4 is a representative two contact device. Device 1 is used to perform polarization and wavelength dependent measurements presented in Fig. 2-3 in the main paper text. Ultrafast measurements are carried out on device 3 (Fig. 5). Device 4 is used for transport and thermoelectric measurements discussed in Fig. 4b. Gate dependent measurements (Fig. 4a) are performed on device 2. It is clear that plasmonic contact of the device 2 has minor defects, while nonplasmonic contact has multilayer regions. Due to these imperfections, a direct comparison of the photovoltage generated for the plasmonic and nonplasmonic contacts in terms of absolute values is complicated. However, the gate dependence of the photovoltage is well preserved, as confirmed by measurements performed on the representative device 4. The results clearly demonstrate that the sign flip occurs at around 25V in good agreement with the trend observed for the nonplasmonic contact of the device 2 (Fig. 4a).


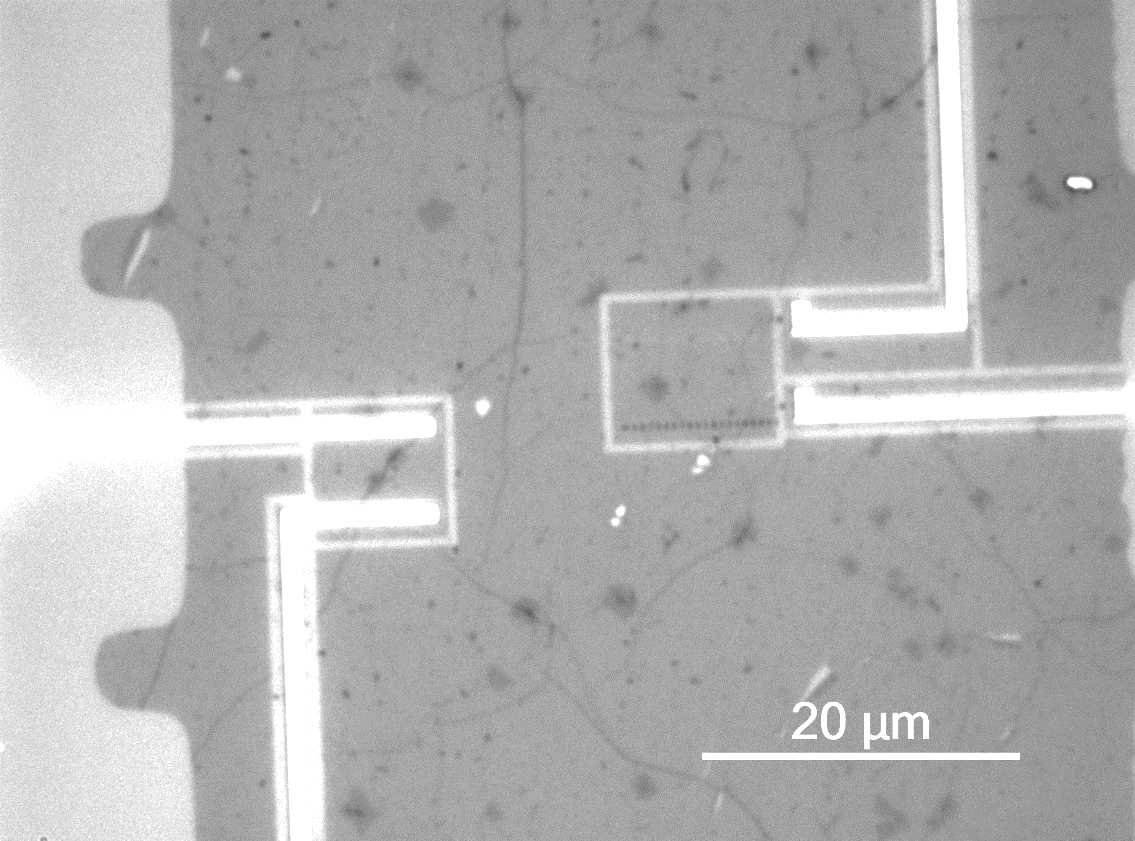


(a)


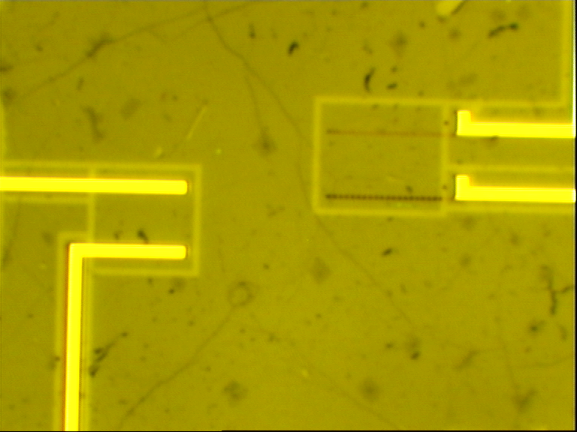

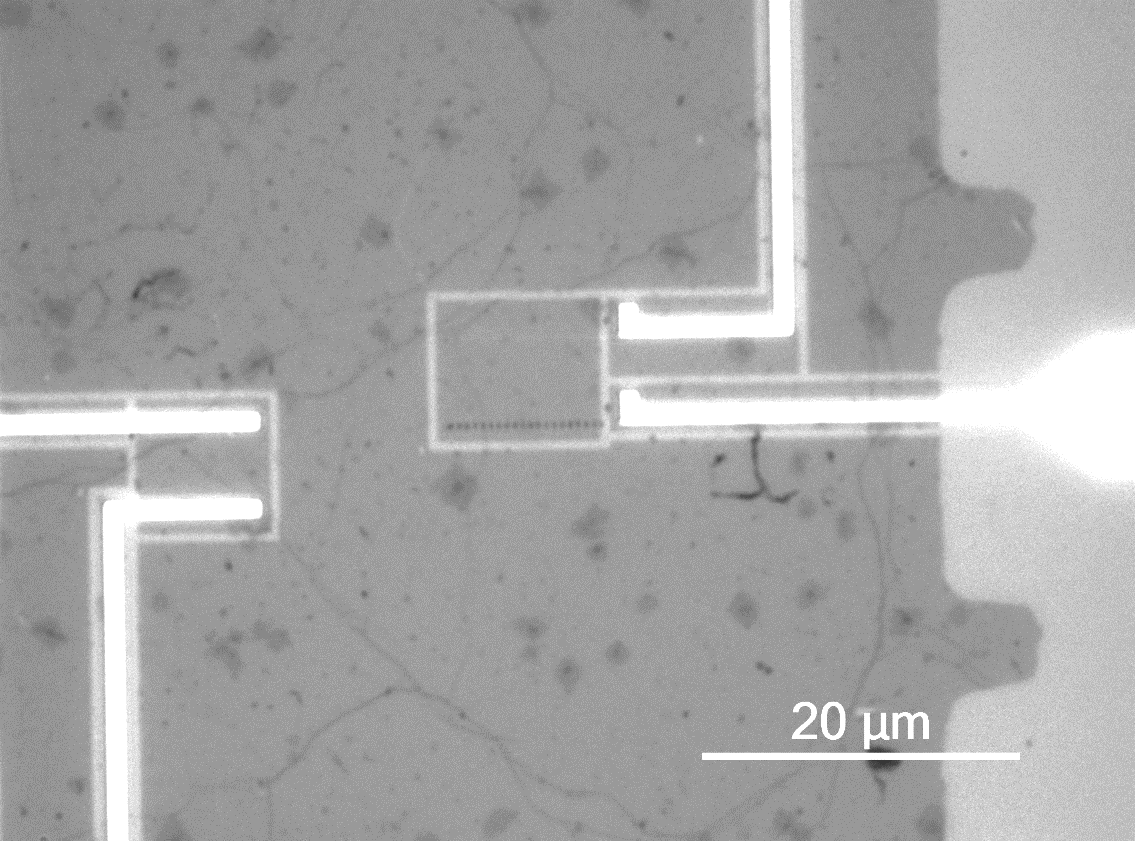

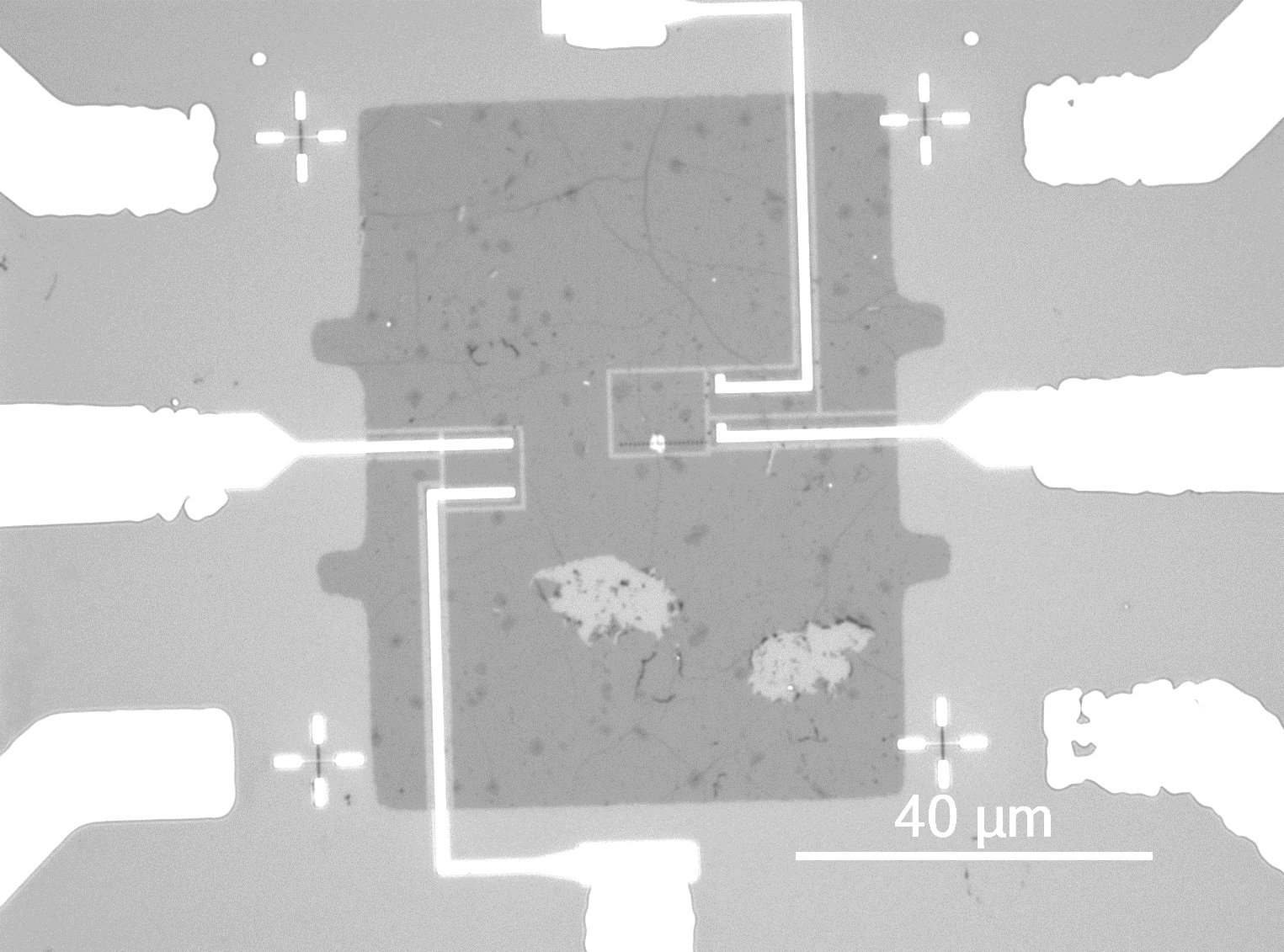


device1

device2

device3

device4

(b)

**Supplementary Figure 3.** (a) Optical microscopy images of the devices tested in the paper. The scale bar is 5μm. (b) Gate dependence of photovoltage for a representative device 4. The photovoltage measurements are performed with elliptical laser spot at 700 nm with 40 µW power. The error bars are the standard deviation in the measurements.

# Supplementary Discussion 4. Graphene quality

## Raman spectroscopy

Supplementary Figure 4 shows the results of Raman spectroscopy, which is utilized to analyse graphene quality and doping concentration in various regions of the device and in particular channel graphene (Gr/SiO_2_) and graphene over gold contact (Gr/Au). Raman spectra confirm the presence of high quality graphene in both regions as evident from low intensity of D-peak. Next, the doping and strain are analysed for the different regions of the sample. It is clear that graphene over gold contact (Gr/Au) exhibits lower doping compared to channel graphene (Gr/SiO_2_), with doping concentration of 2.5∙10^12^ and 5∙10^12^, corresponding to 203 and 287meV for two regions, respectively. These results are in a good agreement with photovoltage measurements discussed in the main text. Notably, the graphene over gold contacts exhibits compressive biaxial strain of -0.05 – 0%, while graphene over substrate SiO_2_ is characterized by tensile biaxial strain of 0 – 0.06%.


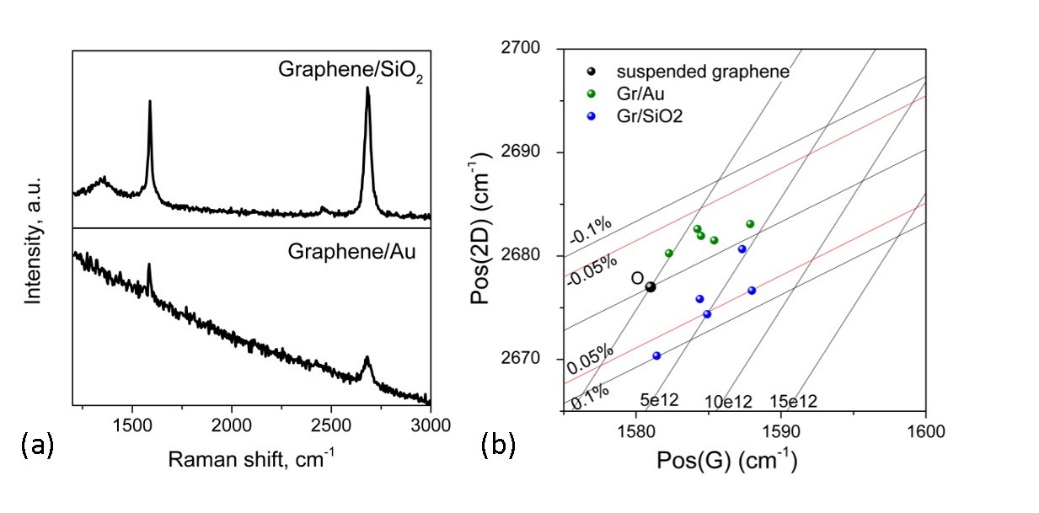


**Supplementary Figure 4.** (a) Representative spectra for Gr/SiO_2_ and Gr/Au areas. (b) Correlation of 2D-peak and G-peak positions. The solid lines of different slope indicate trajectories of equal strain (black and red are used for uniaxial and biaxial strain, respectively) and equal hole doping [3].The indicated doping levels are in cm^-2^. The O point represents suspended exfoliated graphene.

## Atomic Force microscopy

As discussed in the method section, the CVD graphene was transferred on prefabricated contacts. To ensure good material continuity, AFM measurements were carried out. Supplementary Figure 5 shows a typical AFM image of the device with a plasmonic asymmetrical device. It is clear that the graphene film is continuous and not significantly disturbed by the underlying contacts. The measurements were performed in a tapping mode using Veeco Dimension 3100.


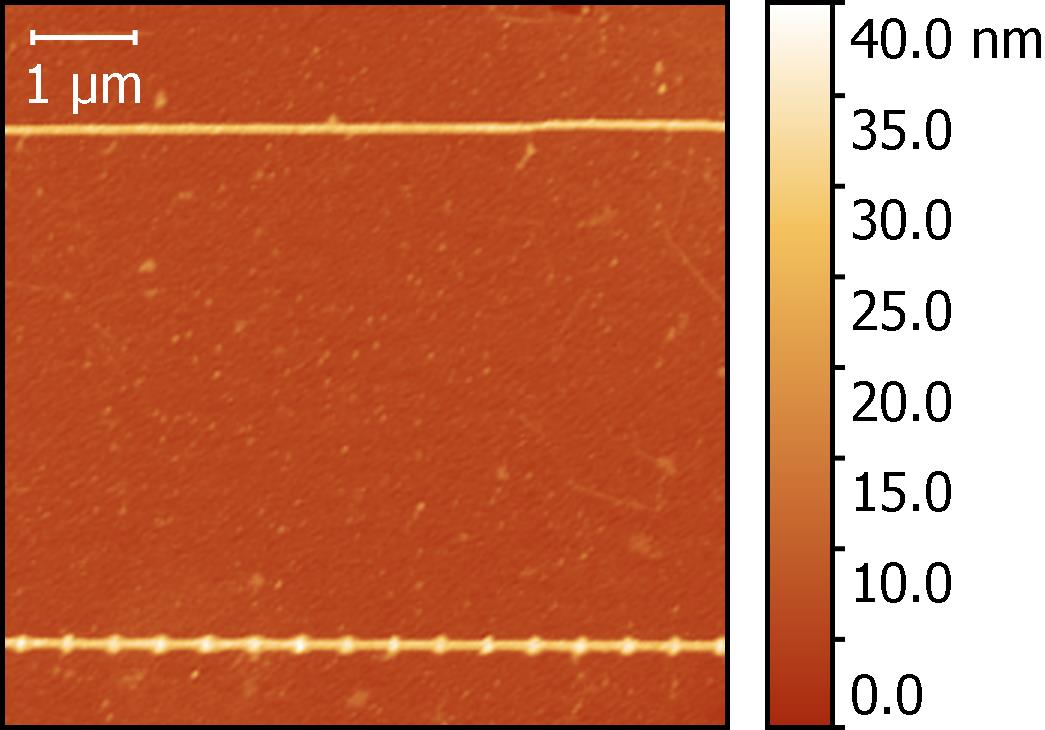


**Supplementary Figure 5.** Representative AFM image of the plasmonic asymmetrical device.

# Supplementary Discussion 5. Conductivity model

To extract the Dirac point position, we compare experimental results with analytical model. The graphene conductivity is modelled using rounded V-shape, as previously described for single layer graphene [4,5]:

| $\sigma=\sigma_{\min}\left( 1+\frac{E_{F}^{4}}{\Delta^{4}} \right)^{\frac{1}{2}}$ | (Supplementary Equation 1) |
| --- | --- |

where σ_min_ is the minimum conductivity and Δ is the width of charge neutrality region, that is related to the disorder strength [6]. This function is expected to correctly describe the graphene conductivity for the highly doped region where σ ~ E_F_^2^ as well as the charge neutrality region where σ ~ constant. Supplementary Figure 6 demonstrates comparison of the experimental data with conductivity models under assumption of Dirac point ($V_{D}$) of 40V and 50V. Clearly, the experimental data is much better described by $V_{D}=40V$ fitting curve with $\sigma= 0.171 mS$ and $\Delta= 0.236 eV$. Thus, we conclude that the Dirac point for our device is ~40V.

**Supplementary Figure 6.** Comparison of experimental and theoretical conductivity. The error bars are the standard deviation in the measurements.

# Supplementary Discussion 6. Seebeck coefficient calculation

As discussed in the main text, Seebeck coefficient ($S$) is a measure of the induced thermoelectric voltage due to a temperature gradient. Graphene’s Seebeck coefficient can be estimated from the material conductivity ($\sigma$) following the Mott relation [7]:

| $S_{Mott}= - \frac{\pi^{2}{k_{B}}^{2}T}{3e}\frac{1}{\sigma}\frac{d\sigma}{dE_{F}}$ | (Supplementary Equation 2) |
| --- | --- |

where $k_{B}$ is the Boltzmann constant, $e$ is electron charge and $E_{F}$ is the chemical potential. The Mott Seebeck coefficient $S_{Mott}$ has been shown to provide a good approximation of $S$ for range of temperatures and carrier densities [8–11]. In fact, Sierra et al. has successfully demonstrated applicability of Mott relation for the device structure where the heater is part of the measurement contacts, which is similar to our device design [10,11]. In these works, Sierra et al. has observed good agreement of Mott Seebeck coefficients with generated thermoelectric voltage for wide range of gate biases and low and room temperature conditions. Additionally, Ghahari et al [12] has recently demonstrated that in low mobility regime, which is applicable to our CVD devices, experimentally extracted Seebeck coefficients exhibit no appreciable deviation from Mott Seebeck coefficients even for room temperatures with only small differences close to the Dirac point. Furthermore, similar approach has been successfully used to describe the photothermoelectric effect in graphene junctions [5,6,13–19].

Using Mott relation, we estimate Seebeck coefficients for both theoretical and experimental conductivity results presented in Supplementary Figure 6. The eq. S2 can be evaluated by replacement of $\frac{d\sigma}{dE_{F}}$ with $\frac{d\sigma}{dV_{g}} \frac{dV_{g}}{dE_{F}}$, where $\frac{d\sigma}{dV_{g}}$ is derived using numerical differentiation of the conductivity *σ* with respect to applied gate bias $V_{g}$. For single layer graphene the dependence of $E_{F}$ on the charge density $n$ is described by $E_{F}=\hbar\nu_{F}\left( \pi n \right)^{\frac{1}{2}}$ where $n=\frac{\varepsilon_{0}\varepsilon_{SiO2}\left( V_{g}-V_{D} \right)}{et}$ and $t$ is the oxide thickness. Therefore, the $\frac{dV_{g}}{dE_{F}}$ term can be described by $\frac{dV_{g}}{dE_{F}}=\frac{2}{\hbar\nu_{F}}\sqrt{\frac{et\left| V_{g}-V_{D} \right|}{{\pi\varepsilon}_{0}\varepsilon_{SiO2}}}$.

Supplementary Figure 7 shows the extracted Seebeck coefficients calculated for the Dirac point of $V_{D}=40V$ and the electronic temperature of $T=300K$. Firstly, we consider the results obtained from the conductivity model introduced in Supplementary Discussion 5 to fit the experimental data (Supplementary Figure 7a). As expected, the sign of the Seebeck coefficient changes across the Dirac point demonstrating the change in the carrier majority and hence the direction of the thermoelectric current. These results are used for the qualitative presentation of different photodetection mechanisms involved in our device, as described in Fig. 1 in the main paper text.

**Supplementary Figure 7.** Seebeck coefficient obtained from the conductivity model as discussed in S4 (a) and from the graphene resistance measurements (b). The right axis in (b) shows calculated $k$ coefficient. The Dirac point of $V_{D}=40V$ and the electronic temperature of $T=300K$ are used.

(a)

(b)

Next, we extract Seebeck coefficient from the experimental conductivity results as presented in Supplementary Figure 7b. The overall shape is reminiscent of typically observed S-shape for graphene’s Seebeck coefficient dependence on the carrier concentration. Furthermore, the extracted values show good correlation with previously measured Seebeck coefficients at room temperature that are typically in the range of 30-70 µV/K [8,9,12,20]. However, it is clear that there are significant discrepancies with the model results which are caused by the conductivity model deviation from the experimentally obtained data as shown in Supplementary Figure 6. Therefore, it is more reasonable to use experimentally obtained Seebeck coefficients to estimate the induced electronic temperature in the device. As discussed in the main paper text, the electronic temperature can be extracted from the experimentally measured photothermoelectric voltage through $T_{C}= \sqrt{{(T_{0})}^{2}+2(\left| V_{PTE-ch}/k \right|)}$, where $k=S/T$ or $k= - \frac{\pi^{2}{k_{B}}^{2}}{3e}\frac{1}{\sigma}\frac{d\sigma}{dE_{F}}$ as derived from the Mott relation (eq. S2). The temperature independent $k$ coefficient is shown as right axis in Supplementary Figure 7b.

# Supplementary Discussion 7. Contribution of the direct laser heating

It is important to analyse the contribution of direct contact heating to the total detector photoresponse. For this purpose, we perform a separate control experiment using a laser to heat one of the metal contacts of a graphene device. An induced difference in temperature between the contacts generates a thermoelectric response originating from the carrier flow in the graphene channel. First, we construct an image of the photoresponse by collecting data as the laser is scanned across the device. Particular attention is paid to the region where the laser excitation is positioned over the metal contacts and local contact heating is introduced. The measurements are performed with a laser power of 40µW, which is the typical average power setting used in the measurements in the main body of work. As clearly seen from the photovoltage map and the cross section taken along a gold contact (Supplementary Figure 8 a-b), the signal generated higher up the contact (dropping away immediately away from the graphene) is at the signal noise level indicating that the thermoelectric contribution due to the increased temperature of a contact is negligible under these illumination conditions.


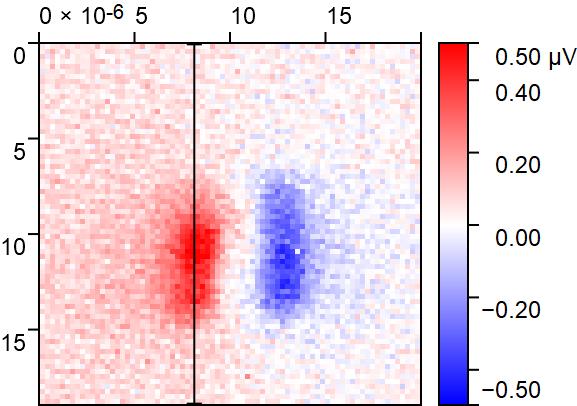


(c)

(a)

(b)

(d)


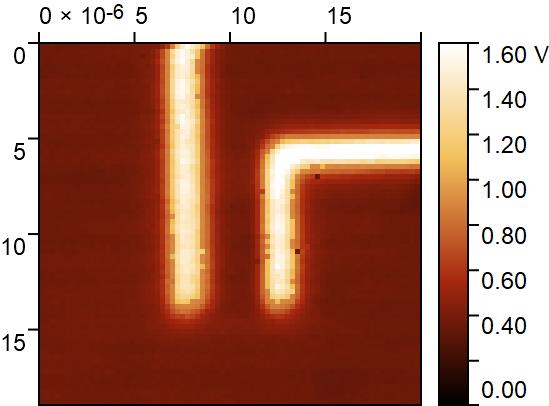


(c)

taken at point x in (c)

µm

µm

**Supplementary Figure 8.** (a, b) Photovoltage map of the device and corresponding cross section for the laser positioned over the left gold contact as indicated by the solid black line. Black dash rectangular shows the position of graphene channel. (c) The corresponding reflection map clearly demonstrating the position of the contact leads. White dash rectangular shows the position of graphene channel. The measurements are performed with focused laser spot of 1 µm for 650 nm excitation wavelength and 40 µW laser power. (d) Power dependence of the induced thermoelectric effect for the laser positioned away from the active graphene area indicated as an x on (c). The error bars are the standard deviation in the measurements.

Next, we position the laser spot away from the active graphene area of the device to examine the influence of a controlled temperature difference by deliberately locally heating one of the contacts as discussed in the main text. It is crucial to position the laser spot away from the active graphene junction to distinguish the thermoelectric effect from any other effects involved in graphene’s photoresponse. The power dependence of the induced thermoelectric voltage is presented in Supplementary Figure 8d. Much higher laser powers were required compared to the main experiments in order to create an observable thermoelectric effect in the sample via direct contact heating. While some enhancement of the thermoelectric response can be expected under specific gate bias conditions, the results presented in Fig. 4b in the main text demonstrate that the generated thermoelectric current remains low with the maximum responsivity of only ~0.2 mV/W observed at ~15V, which is in contrast with the photoresponsivity of our device (~125 mV/W). It should be noted that due to heat dissipation losses, the generated thermoelectric current will increase closer to the graphene junction. However, a previous study by Gabour et al. (see Supplementary information) [19] showed that the thermoelectric voltage induced by direct laser heating increases only slightly near the graphene channel, while much higher photovoltage is registered due to direct graphene absorption. Additionally, the close to uniform temperature across a gold nanowire is further supported by direct measurement of temperature using photoluminescence response of Al_0.94_Ga_0.06_N embedded with Er_3_ by Carlson et al. [21]. Hence, we can conclude that the signals we report in the paper, away from the graphene junction are representative of the direct laser heating effect of gold contact and that at the laser powers used in our experiments, this contribution is negligible.

# Supplementary Discussion 8. Model of the electronic temperature distribution in graphene

As discussed in the main paper text, the strongly enhanced electromagnetic fields in the vicinity of the plasmonic contact create photoexcited carriers in the graphene layer which rapidly thermalize and drastically increase the local electronic temperature. Next, the photoexcited hot carriers diffuse in the graphene layer and create the temperature gradient of the hot carriers in the structure leading to thermoelectric current generation. Here, we analyse the steady-state electronic temperature distribution established in the device under excitation of plasmonic contact. For this purpose, we solve the differential heat transport equation [16,22]:

| $-\kappa_{e}\frac{d^{2}}{{dx}^{2}}T_{e}+\gamma_{e}(x)\left( T_{e}-T_{0} \right)=P\left( x \right),$ | (Supplementary Equation 3) |
| --- | --- |

where $\kappa_{e}$ is the in-plane electronic thermal conductivity, $T_{0}$ is the lattice temperature, which we consider equal to the constant bath temperature of 300K, and $P(x)$ is the excitation profile. Hot carriers in graphene can couple to both internal and substrate phonons. However, the phonon thermal conductivity in graphene is much more efficient leading to negligible lattice temperature increase as discussed in details in Ref. [16,23]. Therefore, the phonon part of the heat conduction is ignored, and the lattice temperature is taken to be at $T_{0}$. To capture the electronic heat dissipation, the out-of-plane thermal conductance $\gamma_{e}$ describing the energy loss to heat sinking substrate is sufficient [16,22]. In this work, we use values of $\gamma_{e}\approx5MW/(m^{2}\cdot K)$ and $\gamma_{e}\approx30MW/(m^{2}\cdot K)$ for the graphene regions on SiO_2_ [24] and Au [25], respectively. The cooling length $\xi=\sqrt{{\kappa_{e}}/{\gamma_{e}}}$ is implemented as a parameter in the range of 0.2-2 µm, consistent with previous studies [5,26].

To solve this equation, we define $\Delta T=T_{e}-T_{0}$, and then the heat transport equation reduces to

| $-\kappa_{e}\frac{d^{2}}{{dx}^{2}}\Delta T+\gamma_{e}\Delta T=P\left( x \right).$ | (Supplementary Equation 4) |
| --- | --- |

Since the local graphene’s absorption is proportional to electromagnetic field intensity $E^{2}(x)$, we firstly extract the local field intensity profile that is then used as $P(x)$ in eq. S4. We calculate the field intensity profile in the plasmonic structure using Lumerical FDTD solutions as presented in Supplementary Figure 9b. To replicate graphene’s behavior around the NPs, the field intensity is recorded on the surface of gold contact and on the surface of the SiO_2_ substrate (Supplementary Figure 9b). For this purpose, two field monitors located at different heights ($z=0 nm$ and $z=40 nm$) are utilized. Next, the Gaussian profile of experimental excitation is implemented through multiplication with $exp(-\left( x-x_{L} \right)^{2}/R_{L}^{2})$, where $x_{L}$ is the position of the laser spot and $R_{L}$ is the laser spot size (in this calculation, we set them as $x_{L}=0 \mu m, and L_{spot}=2\left( 2ln2 \right)^{1/2}R_{L}=1\mu m$).


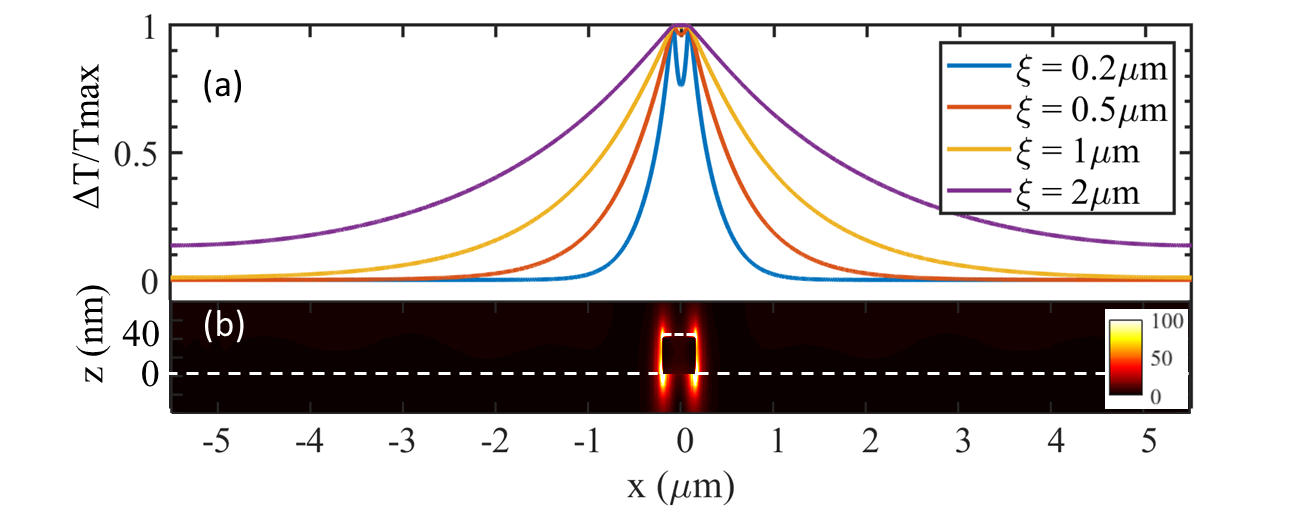


**Supplementary Figure 9.** (a) Electronic temperature profile of the graphene with different cooling lengths (as indicated in the legend) under optical excitation positioned at $x=0 \mu m$. (b) Calculated electromagnetic field distribution for longitudinal polarization at 740 nm. Side view of the structure is displayed. White dash line represents position of the recorded field intensity profile.

We solved eq. S4 numerically to obtain the spatial profile of the electronic temperature as shown in Supplementary Figure 9a for a range of cooling lengths (0.2-5 µm). As expected, a maximum elevated temperature is observed for graphene over the plasmonic contact. For very short cooling length the electronic temperature over the plasmonic nanoparticle is non-monotonic, closely following the electromagnetic field distribution around the structure. For the cooling length of 1 µm, the electronic temperature decays rapidly as expected. In our devices, we estimate the diffusion length of the carriers to be ~700 nm based on the device response time of $\tau_{r}=$ 0.85 ± 0.13 ps and a hot carrier diffusion coefficient of 5500 cm^2^s^-1^ recently measured for CVD graphene [27]. Therefore, the electronic temperature profile obtained for the cooling length of 1 µm can be considered as good approximation for the device steady state operation. It is clear that under these conditions the carrier temperature at the nonplasmonic contact remains low and can be assumed as $T_{0}$ ~ $T_{bath}$ ~ 300 K.

# Supplementary Discussion 9. Calculation of peak photovoltage

The photocarrier dynamics in graphene are well described by a bi-exponential decay [28,29]. Therefore, the photovoltage pulse in graphene devices can be described as following:

| $V_{pulse}\left( t \right)=Ae^{-t/\tau_{1}}+Be^{-t/\tau_{2}}$ | (Supplementary Equation 5) |
| --- | --- |

The peak photovoltage value reached at zero delay time is $V_{pk}=V_{pulse}\left( 0 \right)=A+B$. Under assumption that $B=rA$, the peak value becomes $V_{peak}=A(1+r)$

During the experiment, the average photovoltage is detected that at a repetition rate $\Omega=T^{-1}$ can be expressed as:

$$V_{exp}=\overline{V}_{pulse}=\frac{1}{T}\int_{0}^{T} V_{pulse}dt=\frac{A}{T}\int_{0}^{T} (e^{-t/\tau_{1}}+re^{-t/\tau_{2}})dt=\frac{A}{T}\left( {-\tau_{1}e}^{-\frac{T}{\tau_{1}}}{-r\tau_{2}e}^{-\frac{T}{\tau_{2}}}+\tau_{1}+\tau_{2}r \right)$$

Assuming that $T\gg\tau_{1},\tau_{2}$, $V_{exp}=A(\tau_{1}+\tau_{2}r)/T.$

Eliminating $A$, we find:

| $V_{peak}=V_{exp}\frac{T(1+r)}{\tau_{1}+\tau_{2}r}=V_{exp}\frac{(1+r)}{\Omega\left( \tau_{1}+\tau_{2}r \right)}$ | (Supplementary Equation 6) |
| --- | --- |

If the device response function is described by single exponential decay ($r\mapsto0$), the well-known expression for the peak value is recovered $V_{peak}=V_{exp}\left[ \Omega\tau_{1} \right]^{-1}$, where $\Omega\tau_{1}$ is the laser’s duty cycle. This provides additional justification of the suggested expression.

We can express $V_{peak}$ in eq. S6 through the photovoltage pulse width $\tau_{pulse}$ as following $V_{peak}=V_{exp}\left[ \Omega\tau_{pulse} \right]^{-1}$, where $\tau_{pulse}= \frac{\left( \tau_{1}+\tau_{2}r \right)}{(1+r)}$. Supplementary Figure 10 shows the photovoltage pulse width at different pump laser powers extracted from Fig. 5 presented in the main text of the paper. The average photovoltage pulse width is 0.85±0.13 ps.

**Supplementary Figure 10.** Photovoltage pulse width ($\tau_{pulse}$) under different pump power excitation extracted from Fig. 5 in the main paper text. Error bars reflect the estimated standard deviation of the fit coefficients used to calculate the pulse width.

**Supplementary Figure 11. Photovoltage generation under CW and pulsed excitation**. Measurements are performed at 740 nm wavelength. It is clear that no significant difference is observed. The error bars are the standard deviation in the measurements.

**Supplementary Figure 12. Extended power dependence for plasmonic contact.** Photovoltage generated at 750 nm as a function laser power for the plasmonic contact of the device 2 (Supplementary Discussion 3). The measurements were performed under L-polarization with elliptical beam consistent with the experimental settings used in the main results. The device demonstrates linear photoresponse even for relatively high laser powers.

# Supplementary References:

[1] R. Fernández-García, Y. Sonnefraud, A.I. Fernández-Domínguez, V. Giannini, S.A. Maier, Design considerations for near-field enhancement in optical antennas, Contemp. Phys. 55 (2014) 1–11. doi:10.1080/00107514.2013.850788.

[2] T.G. Habteyes, S. Dhuey, E. Wood, D. Gargas, S. Cabrini, P.J. Schuck, A.P. Alivisatos, S.R. Leone, Metallic adhesion layer induced plasmon damping and molecular linker as a nondamping alternative, ACS Nano. 6 (2012) 5702–5709. doi:10.1021/nn301885u.

[3] J.E. Lee, G. Ahn, J. Shim, Y.S. Lee, S. Ryu, Optical separation of mechanical strain from charge doping in graphene., Nat. Commun. 3 (2012) 1024. doi:10.1038/ncomms2022.

[4] X. Cai, A.B. Sushkov, R.J. Suess, M.M. Jadidi, G.S. Jenkins, L.O. Nyakiti, R.L. Myers-Ward, S. Li, J. Yan, D.K. Gaskill, T.E. Murphy, H.D. Drew, M.S. Fuhrer, Sensitive room-temperature terahertz detection via the photothermoelectric effect in graphene., Nat. Nanotechnol. 9 (2014) 814–9. doi:10.1038/nnano.2014.182.

[5] R.-J. Shiue, Y. Gao, Y. Wang, C. Peng, A.D. Robertson, D.K. Efetov, S. Assefa, F.H.L. Koppens, J. Hone, D. Englund, High-Responsivity Graphene–Boron Nitride Photodetector and Autocorrelator in a Silicon Photonic Integrated Circuit, Nano Lett. 15 (2015) 7288–7293. doi:10.1021/acs.nanolett.5b02368.

[6] J.C.W. Song, M.S. Rudner, C.M. Marcus, L.S. Levitov, Hot carrier transport and photocurrent response in graphene., Nano Lett. 11 (2011) 4688–92. doi:10.1021/nl202318u.

[7] M. Cutler, N.F. Mott, Observation of anderson localization in an electron gas, Phys. Rev. 181 (1969) 1336–1340. doi:10.1103/PhysRev.181.1336.

[8] Y.M. Zuev, W. Chang, P. Kim, Thermoelectric and magnetothermoelectric transport measurements of graphene., Phys. Rev. Lett. 102 (2009) 096807. doi:10.1103/PhysRevLett.102.096807.

[9] P. Wei, W. Bao, Y. Pu, C.N. Lau, J. Shi, Anomalous thermoelectric transport of Dirac particles in graphene., Phys. Rev. Lett. 102 (2009) 166808. doi:10.1103/PhysRevLett.102.166808.

[10] J.F. Sierra, I. Neumann, J. Cuppens, B. Raes, M. V Costache, S.O. Valenzuela, Thermoelectric spin voltage in graphene, Nat. Nanotechnol. 13 (2018) 107–112. doi:10.1038/s41565-017-0015-9.

[11] J.F. Sierra, I. Neumann, M. V Costache, S.O. Valenzuela, Hot-Carrier Seebeck Effect: Diffusion and Remote Detection of Hot Carriers in Graphene., Nano Lett. (2015). doi:10.1021/acs.nanolett.5b00922.

[12] F. Ghahari, H.-Y. Xie, T. Taniguchi, K. Watanabe, M.S. Foster, P. Kim, Enhanced Thermoelectric Power in Graphene: Violation of the Mott Relation by Inelastic Scattering, Phys. Rev. Lett. 116 (2016) 136802. doi:10.1103/PhysRevLett.116.136802.

[13] T.J. Echtermeyer, P.S. Nene, M. Trushin, R. V. Gorbachev, A.L. Eiden, S. Milana, Z. Sun, J. Schliemann, E. Lidorikis, K.S. Novoselov, A.C. Ferrari, Photothermoelectric and Photoelectric Contributions to Light Detection in Metal–Graphene–Metal Photodetectors, Nano Lett. 14 (2014) 3733–3742. doi:10.1021/nl5004762.

[14] M.C. Lemme, F.H.L. Koppens, A.L. Falk, M.S. Rudner, H. Park, L.S. Levitov, C.M. Marcus, Gate-activated photoresponse in a graphene p-n junction., Nano Lett. 11 (2011) 4134–7. doi:10.1021/nl2019068.

[15] X. Xu, N.M. Gabor, J.S. Alden, A.M. van der Zande, P.L. McEuen, Photo-thermoelectric effect at a graphene interface junction., Nano Lett. 10 (2010) 562–6. doi:10.1021/nl903451y.

[16] M. Freitag, T. Low, P. Avouris, Increased responsivity of suspended graphene photodetectors., Nano Lett. 13 (2013) 1644–8. doi:10.1021/nl4001037.

[17] E.C. Peters, E.J.H. Lee, M. Burghard, K. Kern, Gate dependent photocurrents at a graphene p-n junction, Appl. Phys. Lett. 97 (2010) 193102. doi:10.1063/1.3505926.

[18] M. Freitag, T. Low, F. Xia, P. Avouris, Photoconductivity of biased graphene, Nat. Photonics. 7 (2012) 53–59. doi:10.1038/nphoton.2012.314.

[19] N.M. Gabor, J.C.W. Song, Q. Ma, N.L. Nair, T. Taychatanapat, K. Watanabe, T. Taniguchi, L.S. Levitov, P. Jarillo-Herrero, Hot carrier-assisted intrinsic photoresponse in graphene., Science. 334 (2011) 648–52. doi:10.1126/science.1211384.

[20] A. Sherehiy, R. Jayasinghe, R. Stallard, G. Sumanasekera, A. Sidorov, D. Benjamin, Z. Jiang, Q. Yu, W. Wu, J. Bao, Z. Liu, S. Pei, Y. Chen, Thermoelectric properties of CVD grown large area graphene, Am. Phys. Soc. APS March Meet. 2010, March 15-19,2010, Abstr. #Z21.004. (2010). http://adsabs.harvard.edu/abs/2010APS..MARZ21004S (accessed August 9, 2017).

[21] M.T. Carlson, A.J. Green, A. Khan, H.H. Richardson, Optical measurement of thermal conductivity and absorption cross-section of gold nanowires, J. Phys. Chem. C. 116 (2012) 8798–8803. doi:10.1021/jp212575a.

[22] A. Woessner, P. Alonso-González, M.B. Lundeberg, Y. Gao, J.E. Barrios-Vargas, G. Navickaite, Q. Ma, D. Janner, K. Watanabe, A.W. Cummings, T. Taniguchi, V. Pruneri, S. Roche, P. Jarillo-Herrero, J. Hone, R. Hillenbrand, F.H.L. Koppens, Near-field photocurrent nanoscopy on bare and encapsulated graphene, Nat. Commun. (2016) 1–7. doi:10.1038/ncomms10783.

[23] M. Freitag, M. Steiner, Y. Martin, V. Perebeinos, Z. Chen, J.C. Tsang, P. Avouris, Energy Dissipation in Graphene Field-Effect Transistors, Nano Lett. 9 (2009) 1883–1888. doi:10.1021/nl803883h.

[24] T. Low, V. Perebeinos, R. Kim, M. Freitag, P. Avouris, Cooling of photoexcited carriers in graphene by internal and substrate phonons, Phys. Rev. B - Condens. Matter Mater. Phys. 86 (2012) 1–9. doi:10.1103/PhysRevB.86.045413.

[25] W. Cai, A.L. Moore, Y. Zhu, X. Li, S. Chen, L. Shi, R.S. Ruoff, Thermal transport in suspended and supported monolayer graphene grown by chemical vapor deposition, Nano Lett. 10 (2010) 1645–1651. doi:10.1021/nl9041966.

[26] Q. Ma, N.M. Gabor, T.I. Andersen, N.L. Nair, K. Watanabe, T. Taniguchi, P. Jarillo-Herrero, Competing Channels for Hot-Electron Cooling in Graphene, Phys. Rev. Lett. 112 (2014) 247401. doi:10.1103/PhysRevLett.112.247401.

[27] B.A. Ruzicka, S. Wang, J. Liu, K.-P. Loh, J.Z. Wu, H. Zhao, Spatially resolved pump-probe study of single-layer graphene produced by chemical vapor deposition [Invited], Opt. Mater. Express. 2 (2012) 708. doi:10.1364/OME.2.000708.

[28] A. Urich, K. Unterrainer, T. Mueller, Intrinsic response time of graphene photodetectors, Nano Lett. 11 (2011) 2804–2808. doi:10.1021/nl2011388.

[29] K.J. Tielrooij, L. Piatkowski, M. Massicotte, A. Woessner, Q. Ma, Y. Lee, K.S. Myhro, C.N. Lau, P. Jarillo-Herrero, N.F. van Hulst, F.H.L. Koppens, Generation of photovoltage in graphene on a femtosecond timescale through efficient carrier heating, Nat. Nanotechnol. 10 (2015) 437–443. doi:10.1038/nnano.2015.54.
